# Keeping children within the family: Changing patterns in the use of special guardianship orders

**DOI:** 10.23889/ijpds.v8i2.2231

**Published:** 2023-09-14

**Authors:** Bachar Alrouh, Judith Harwin

**Affiliations:** 1 Lancaster University, Lancaster, United Kingdom

Special Guardianship Orders (SGOs) provide family courts with an alternative to adoption, typically through permanent placement within the extended family. Using court administrative data, this study charts the use of SGOs as a way of promoting stability and recovery for these vulnerable children who cannot remain safely with their parents.

The study cohort is based on administrative population data produced routinely by Cafcass and accessed via the Secure Anonymised Information Linkage (SAIL) Databank at Swansea University. The unit of analysis is children subject to s.31 care and supervision proceedings in England between 2007/08 and 2021/22, providing a retrospective observational window of fifteen years. Descriptive statistics were used to compare SGOs to other permanency final legal orders (placement, child arrangements, supervision and no order), and survival modelling was employed to look at stability over time and return to court. Finally, regional and over time variations in the data were examined.

The percentage of children placed on a SGO as a proportion of all children subject to s.31 proceedings rose from 12% to 18% between 2011/12 and 2021/22. Over the same period the percentage of children with a placement order that frees the way for adoption fell from 24% to 14%. This shift in pattern was more pronounced than for any other legal order we examined for children unable to remain with birth parents. The highest usage per region was in the North East and London and the lowest was in the Midlands. We estimate that 9% of children on SGOs would return to court within 10 years. Older children (5-17 years old) were more likely to return to court after SGOs than younger children.

The study showed that SGOs have become a main route out of public care, outstripping the use of placement orders. These findings have major implications for policy and practice. They demonstrate that special guardianship provides a stable and sustainable permanency option for children unable to remain with their birth parents.

